# A Novel Liposomal In-Situ Hydrogel Formulation of *Hypericum perforatum* L.: In Vitro Characterization and In Vivo Wound Healing Studies

**DOI:** 10.3390/gels11030165

**Published:** 2025-02-26

**Authors:** Ahmet Arif Kurt, İsmail Aslan

**Affiliations:** 1 Department of Pharmaceutical Technology, Faculty of Pharmacy, Suleyman Demirel University, Isparta 32000, Türkiye; 2 Department of Pharmaceutical Technology, Hamidiye Faculty of Pharmacy, University of Health Sciences, Istanbul 34668, Türkiye; ismail.aslan@sbu.edu.tr; 3 Faculty of Pharmacy, Istanbul Kent University, Istanbul 34406, Türkiye

**Keywords:** liposomes, hydrogels, in situ polymers, wound healing, *Hypericum perforatum* L.

## Abstract

*Hypericum perforatum* L. (H.P.) is a species with a well-documented history of use in wound healing practices across the globe. The objective of this study was twofold: firstly, to evaluate the in vivo efficacy of liposomal in situ gel formulations in wound healing, both clinically and histopathologically, and secondly, to determine the physicochemical characterization of liposomal in situ gel formulations. The in vitro studies will be assessed in terms of particle size, zeta potential, release kinetics, rheological behaviors, and antioxidant and antimicrobial properties. The in vivo studies will be evaluated in clinical animal experiments and pathology studies. The in-situ hydrogel formulations were prepared using the physical cross-linking method with Poloxamer 188, Poloxamer 407, Ultrez 21, and Ultrez 30. The liposome formulations phospholipid 90H and lipoid S100 were prepared using the thin film solvent evaporation method. The antioxidant activity of the samples was evaluated through in vitro studies employing the DPPH antioxidant activity, ABTS+ test, and FRAP test. The antimicrobial activity of the samples was evaluated through the determination of MIC and MBC values employing the 96-well plate method. In vivo, 36 male New Zealand rabbits aged 32–36 weeks were utilized, with six rabbits in each group. The groups were composed of six distinct groups, including conventional and in situ gel liposome formulations of HHPM, three different commercial preparations, and a control group (*n* = 6). The HHPM-LG8 formulation developed in this study was found to be applicable in terms of all its properties. The new liposomal in situ hydrogel formulation demonstrated notable wound healing activity, a result that was supported by the formulation itself.

## 1. Introduction

The utilization of plants in a variety of domains has been documented, ranging from aesthetic applications to medicinal treatments and cosmetic care [1,2,3,4,5]. One of the herbal active ingredients, hypericin, found in the oil extracted by maceration of the *Hypericum perforatum* plant [6,7], has been shown to possess anti-inflammatory properties [8] and to promote wound healing [9].

In order to maintain the effectiveness of herbal actives in the target area, it may be necessary to encapsulate them using suitable carrier systems [10,11,12]. In this field, liposome carrier systems have been utilized in the encapsulation of herbal actives and macerates [13,14,15,16].

Liposomes can be regarded as synthetic reproductions of natural membranes. These particles, which comprise phospholipids, cholesterol, and a variety of transport donors within their structure, bear a strong resemblance to cell membranes and consequently exhibit a high degree of compatibility with biological systems [17]. Liposomes, which are the focus of extensive research in contemporary scientific discourse, are predominantly spherical in shape and comprise polar lipid layers and an aqueous region between these layers. The dimensions of liposomes range from nanometers to micrometers [18]. In accordance with their physicochemical properties, liposomes are capable of transporting molecules of significance in the fields of pharmaceuticals and food production to disparate regions [19]. In a manner analogous to cells in biological systems, liposomes have the capacity to retain hydrophilic drug active ingredients in their internal regions and active ingredients with high-fat solubility and amphiphilic molecules in their membrane parts. The utilization of liposomal carrier systems in both therapeutic and diagnostic fields is favored due to their numerous advantages. In addition to being biodegradable, liposomal carrier systems are bio-permeable and biocompatible [20]. The high degree of biocompatibility of these systems is attributable to their incorporation within the structure of biological membranes [21]. The low systemic toxicity and toxicity to cells is a consequence of their high degree of biocompatibility [22]. The presence of hydrophilic and lipophilic regions within the liposomal structure facilitates the transportation of both hydrophilic and lipophilic drug substances [23]. The controlled and sustained release of drugs can be achieved through the utilization of liposomal carrier systems [24]. Furthermore, the controlled release of the drug ensures that the plasma drug concentration can be maintained at the desired level for the desired period of time [25]. From a therapeutic standpoint, extended-release liposomal systems have been shown to reduce toxic effects while simultaneously extending the dosing interval and prolonging the drug’s half-life [26].

Gels are defined as transparent or translucent, non-greasy, semisolid formulations. They are considered to be an excellent form for several routes of administration, including oral, topical, vaginal, and rectal delivery [27]. It is noteworthy that gels can be characterized as transparent formulations when all of the particles completely dissolve in the dispersing medium [28]. However, this phenomenon does not occur in all gels, and some are, therefore, turbid. It is, therefore, evident that clear gels are preferred by patients [29]. Gels are also defined as semisolid systems comprising a liquid phase that is confined within a 3D polymeric matrix [30]. The matrix is composed of natural or synthetic gum, and it exhibits a high degree of physical or chemical cross-linking [31]. The formation of gels is facilitated by the utilization of gelling agents, which undergo a significant degree of cross-linking or association upon hydration and dispersion in the medium used for their dispersion or when dissolved in the said medium [32].

A plethora of gelling agents are available [33]. The most common include acacia, alginic acid, bentonite, carbomers, carboxymethylcellulose, ethylcellulose, gelatin, hydroxyethylcellulose, hydroxypropyl cellulose, poloxamers, polyvinyl alcohol, sodium alginate, tragacanth, and xanthan gum [34]. Though each gelling agent has some unique properties, there are some generalizations that can be made.

Carbomer is a generic designation for a family of polymers known as Carbopol^®^ [35]. The first documented use of Carbopol^®^ polymers occurred in the mid-1950s [36]. These polymers are characterized by their dry powder composition and high bulk densities, and they form acidic aqueous solutions with a pH of approximately 3.0. They thicken at higher pHs (around 5 or 6). They also exhibit significant swelling in aqueous solutions of that pH, reaching up to 1000-fold their original volume. The viscosity of their solutions ranges from 0 to 80,000 centipoise (cps).

Poloxamer is a class of copolymers of polyoxyethylene (POE) and polyoxypropylene (PPO) [37]. They have the capacity to form thermoreversible gels in concentrations ranging from 15% to 50%. This indicates that they are liquid at low temperatures (i.e., refrigerator temperature) but gel-like at room or body temperature [38].

Liposomes, comprising an internal gelatinized nucleus, suspended in an aqueous medium and containing low concentrations of gelatinizing substances, can be designated “liposomal in-situ hydrogels”. A process for manufacturing such liposomes or liposomal hydrogels has been developed in which the encapsulated aqueous phase is present in semi-solid gel form and not in liquid form, thereby preventing the liposomes from fusing when collisions occur [39]. A salient feature of these liposomal hydrogels is their production entirely from natural substances, a factor that serves to minimize the risk of adverse reactions due to intolerance [40]. The liposomal hydrogels are constituted of a single bilayer interfacial phase in the case of unilamellar liposomal hydrogels or a multitude of bilayer interfacial phases that are superimposed concentrically in the case of multilamellar liposomal hydrogels [41]. Additionally, a gelatinized encapsulated internal aqueous polar phase is present, wherein the gelatinized substance, which may or may not be polymerizable, is selected from polysaccharides, polypeptides, or 18 polyacrylamides [42]. For instance, the non-polymerizable gelatinizable substance is selected from gelatin, agarose, or carrageenans, while the polymerizable gelatinizable substance is selected from polyacrylamide gels. The liposomal hydrogels thus obtained are characterized by enhanced stability, a significant improvement on the stability of the prior art liposomes, attributable chiefly to the absence of interparticulate fusion during collisions [43]. In typical circumstances, the capacity of liposome carrier systems to bind to the application surface is minimal. The incorporation of hydrogels comprising a secondary carrier system, such as poloxamer and carbomer-type gels, results in the formation of a steric barrier for liquids, including liposomes. This augmentation in adhesion to surfaces is consequent to the steric hindrance. Furthermore, the utilization of hydrogels has the potential to extend the duration of the treatment and enhance its efficacy through the regulated release of the active constituent. Consequently, as an alternative to conventional wound care products, innovative and effective formulations that will further accelerate wound healing may be designed.

This research study contains in vitro characterization and in vivo wound healing studies of liposomal in-situ hydrogel formulation of *Hypericum perforatum* L.

## 2. Results and Discussion

### 2.1. Particle Size, Polydispersity Index, Zeta Potential, and Encapsulation Efficiency

The particle size, PDI (Polydispersity index), zeta potential, and encapsulation efficiency are determinant factors; the choice of the optimum liposome formulation and *H. perforatum* L. macerate (HHPM)-loaded liposome dispersions are given in Table 1 (n = 3). Analyses of the amounts of constituents in the HHPM content were carried out according to the method of the previous studies and according to the ICH Q2 R1 guideline [43]. The encapsulation efficiencies of HHPM-loaded liposomes were HP-LG1 (84.22%/40.992 µg), HP-LG2 (89.35%/43.489 µg), HP-LG3 (85.79%/41.756 µg), and HP-LG4 (91.83%/44.696 µg).

### 2.2. HPLC Result

**Figure 1:**
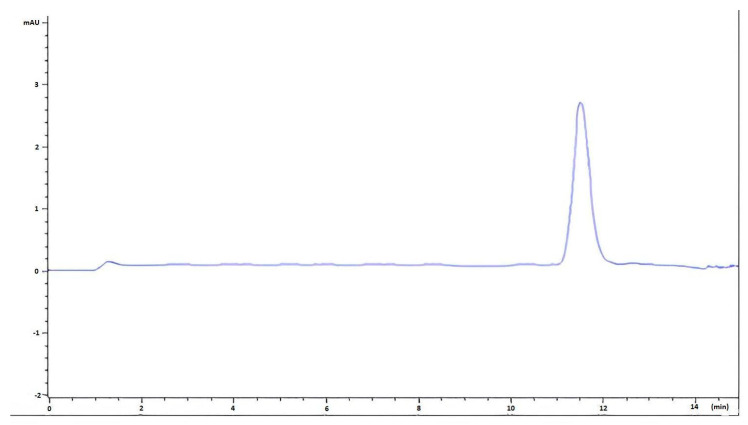
HPLC chromatograms of hypericin in *H. perforatum* L. macerate-containing liposome sample (HHPM-L4).

Evaluating these results reveals that the more effective drug loading capacity of the HHPM-L4 formulation is critical in deciding on the optimum liposome formulation. The peak graph obtained from the HPLC of HHPM-L4 is given in Figure 1, and the amount of hypericin content was found to be 0.487%.

### 2.3. SEM Image Results

**Figure 2:**
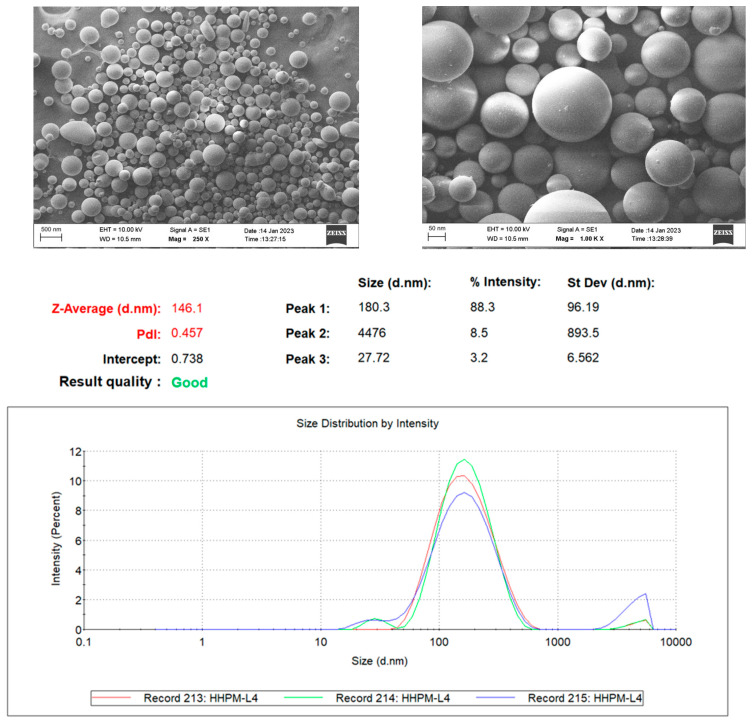
HPM-L4 SEM and DLS images.

SEM images were obtained to ascertain the shape of the liposomes in the optimum formulation, HHPM-LG4, and it was established that they generally have a uniform spherical shape. SEM images of HHPM-LG4 show that the particle sizes are mostly homogeneous (Figure 2), and the particle size is below 200 nm at the nanoscale (Figure 2).

### 2.4. Rheological Behavior

**Figure 3:**
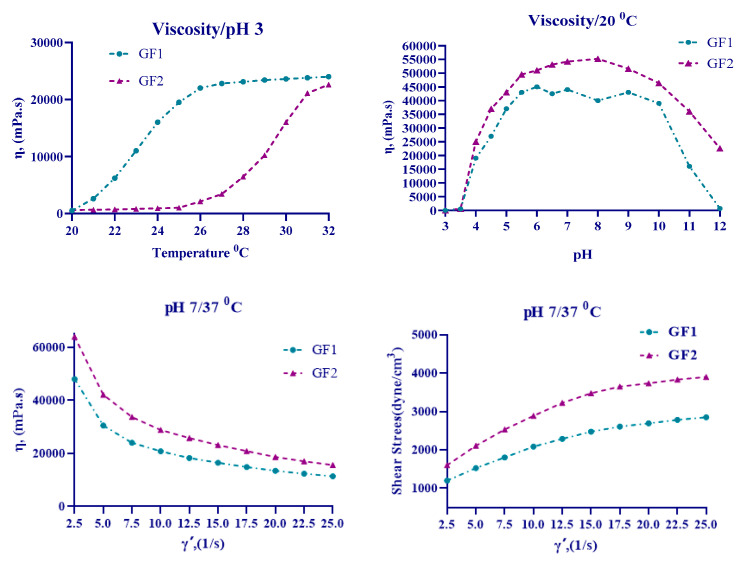
The in-situ gels’ rheological behavior at different pH and temperatures of GF1 and GF2.

A comprehensive analysis was conducted on the rheological properties of carbomer derivatives containing in-situ hydrogel formulations GF1 (Poloxamer 188, Poloxamer 407, and U21 containing in-situ hydrogel) and GF2 (Poloxamer 188, Poloxamer 407, and U30 containing in-situ hydrogel). This analysis encompassed the measurement of viscosity and shear stress responses, along with the examination of the alterations in viscosity and shear stress of gelling agents U-21 and U-30 across a range of pH values. Additionally, the temperature-dependent changes in viscosity of Poloxamer 407 and Poloxamer 188 were investigated. The rheological behavior images of in-situ hydrogel formulations at varying pH and temperature values are illustrated in Figure 3.

It was demonstrated that both formulations, GF1 and GF2, exhibited pseudoplastic flow, which is a non-Newtonian flow type characterized by shear-thinning behavior.

### 2.5. Kinetic Studies

**Figure 4:**
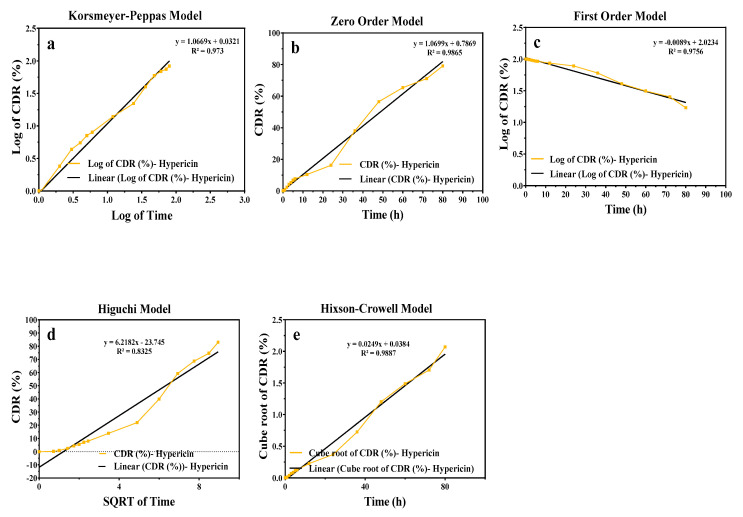
The different release kinetic models of the HHPM-LG8. (**a**) Korsmeyer Peppas (**b**) Zero Order (**c**) First Order (**d**) Higuchi (**e**) Hixson-Crowell.

The release kinetics of the active component from the HHPM L-4 shows generated Korsmeyer-Peppas, zero-order, first-order, Higuchi, and Hixson–Crowell kinetic models generated with data obtained from drug release studies (Table 2 and Figure 4).

The release kinetics studies of the accumulated total active substance (hypericin) against time are demonstrated in Figure 4. Based on the r^2^ value, it was determined that the release of *H. perforatum* L. macerate from HHPM-LG8 in-situ liposome was in accordance with the Hixson–Crowell model.

### 2.6. Gel Formulation Stabilities at pH 3 and pH 7

The stability of the gel formulations at pH 3 and pH 7 was examined over a 180-day period. The ambient conditions were maintained at a constant temperature of 4 ± 2 °C, and the alterations in rheological and organoleptic properties of the gels in response to pH fluctuations were meticulously observed and documented in Table 3.

### 2.7. In Vitro Release Studies

**Figure 5:**
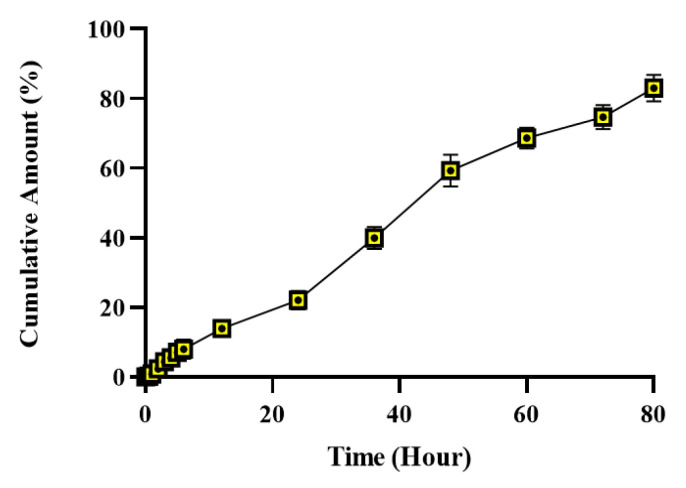
Graph of % cumulative hypericin content from HHPM-LG8 versus time.

The in vitro release study was performed using HPLC at 270 nm, employing the dialysis bag method. The results of the measurements made from the samples taken at 0, 5, 1, 2, 3, 4, 5, 6, 12, 24, 36, 60, 72, and 80 h are shown in Figure 5 as % cumulative hypericin content versus time. As demonstrated in the graph, the controlled release is sustained until the 10th hour. At the conclusion of the 80th hour, the calculated release was determined to be 83.03%.

### 2.8. Stability Findings of Liposomal In Situ Hydrogel formulations

Following the conduction of stability studies, the HHPM-LG8 formulation was determined to be the most appropriate formulation as a consequence of the findings from long-term and real-time stability studies. Stability studies were conducted for HHPM-LG8 at temperatures of 4 ± 2 °C and 25 ± 2 °C, in a relative humidity range of 60 ± 5%, over a period of 180 days. The results of these studies are presented in Table 4 and Table 5.

### 2.9. Antioxidant Activity

**Figure 6:**
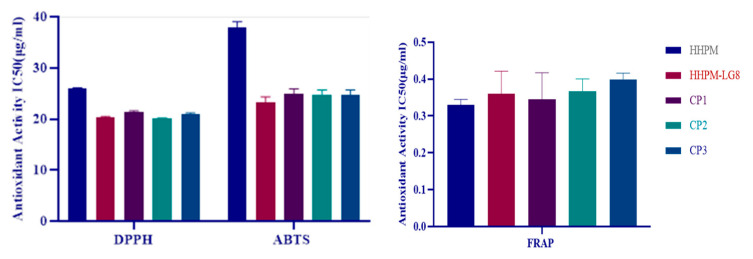
DPPH, ABTS, and FRAP antioxidant activity results.

Antioxidant activity results were given in Figure 6. The results demonstrate that the following substances were effective in inhibiting DPPH (2,2-diphenyl-1-picrylhydrazyl) radicals at an IC50 concentration: HHPM, HHPM-LG8, CP1 (a commercial product containing hypericin), and CP2 (a commercial product containing madecassoside). The IC50 (μg/mL) values for CP3 (polymyxin B containing commercial product) were found to be 24.016 ± 0.101 μg/mL, 20.308 ± 0.207 μg/mL, 21.317 ± 0.313 μg/mL, 21.022 ± 0.203 μg/mL, and 20.134 ± 0.113 μg/mL, respectively. The developed formulations demonstrate comparable levels of antioxidant activity.

The ability to inhibit ABTS (2,2′-azino-bis(3-ethylbenzothiazoline-6-sulfonic acid) radicals was investigated, with the IC50 (μg/mL) value for HHPM, HHPM-LG8, CP1, CP2, and CP3 being 38.016 ± 1.024. μg/mL, 23.205 ± 1.189 μg/mL, 24.901 ± 1.038 μg/mL, 24.819 ± 0.887 μg/mL, and 22.809 ± 0.754 μg/mL, respectively.

The ferric ion reducing antioxidant power of the HHPM, HHPM-LG8, CP1, CP2, and CP3 products was 0.3298 ± 0.015 mM TE/g, 0.3603 ± 0.061 mM TE/g, 0.3452 ± 0.072 mM TE/g, 0.3676 ± 0.033 mM TE/g, and 0.3992 ± 0.017 mM TE/g, respectively.

### 2.10. Microdilution Method

The minimum inhibitory concentration (MIC) and minimum bacteriostatic concentration (MBC) values of *H. perforatum* L. (St. John’s Wort) macerates containing liposome in-situ hydrogel formulations determined according to the microdilution method. The MIC and MBC activity against *C. albicans* and *E. coli* strains are shown in Table 6.

The study showed that liposome in-situ gel may have antibacterial activity at lower concentrations. When the MIC results were analyzed, it was observed that the liposome in-situ gel formulation showed higher activity in *E. coli* and *C. albicans* strains with a concentration of 1.25% compared to CP1 and CP2. CP 3 was already an antibiotic-sourced commercial product. In addition, *C. albicans* and *E. coli* strains showed less sensitivity than others.

### 2.11. In Vivo Clinical Findings

**Figure 7:**
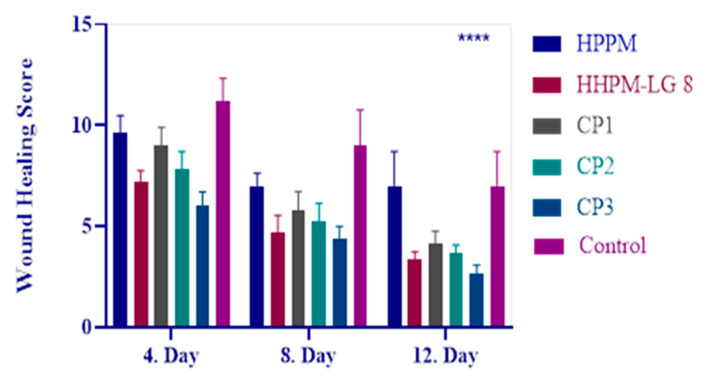
Wound healing score of HHPM, HHPM-LG8, CP1, CP2, and CP3 in the skin defect area after 4, 8, and 12 days. **** means *p* < 0.0001.

**Figure 8:**
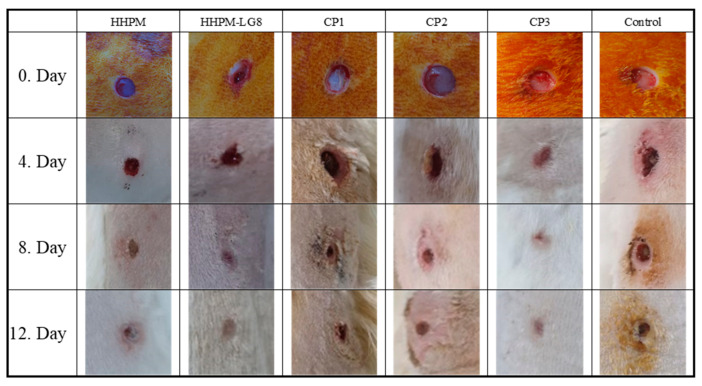
Wound healing images of HHPM, HHPM-LG8, CP1, CP2, and CP3 in the skin defect area after 0, 4, 8, and 12 days.

The results show that there are significant differences in wound healing scores between the groups on days 4, 8, and 12 compared to the control group (*p* < 0.05). The efficacy of HHPM was found to be low regarding wound healing, but the liposomal in-situ gel formulation HPPM-LG8 was identified as one of the most effective formulations. A comparison of the wound closure images demonstrates the effectiveness of the formulation. The commercial preparations CP1 and CP2 showed a lower efficacy despite containing herbal content. The findings underscore the significance of the liposomal in-situ gel formulation in potentiating the efficacy of HHPM. This indicates that the treatments had a significant effect on wound healing compared to no treatment (Figure 7 and Figure 8).

### 2.12. Histopathological Findings

**Figure 9:**
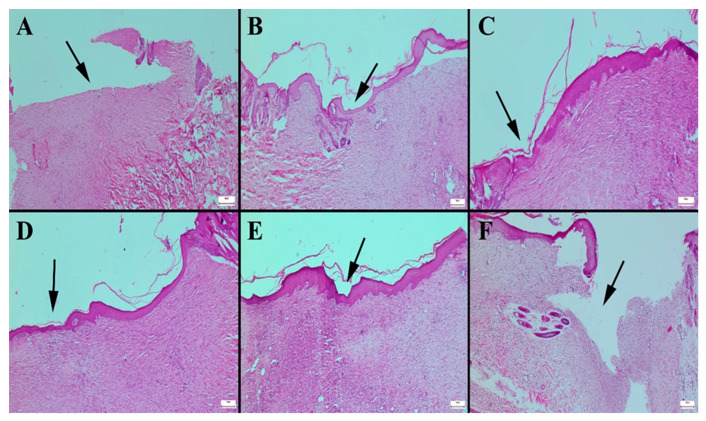
Histopathological appearance of wound healing in the skin defect area according to groups. (**A**) HHPM, (**B**) HHPM-LG8, (**C**) CP1, (**D**) CP2, (**E**) CP3, (**F**) Control; Bars = 50 µm.

Histopathological analysis of the skin defect areas revealed significant differences in the healing response among the treatment groups. Epithelialization was notably improved in the group treated with the HHPM-LG8 formulation, as evidenced by a well-formed epithelial layer covering the wound site (Figure 9B). The extent of epithelialization observed in this group was comparable to that of commercial products CP1 and CP2, both of which are recognized for their effectiveness in promoting wound healing (Figure 9C,D). In contrast, the untreated control group exhibited the slowest healing process, characterized by incomplete epithelialization, minimal collagen synthesis, and limited angiogenesis. These findings indicate a delayed and inefficient wound-healing response, with inadequate tissue regeneration and insufficient vascularization to support new tissue formation (Figure 9F). Treatment with HHPM alone resulted in only moderate healing effects, with partial epithelialization and a lower degree of collagen deposition compared to HHPM-LG8. Although some improvement was observed, the overall wound closure process remained suboptimal (Figure 9A). Among all formulations tested, CP3 demonstrated the most pronounced healing effects. Histopathological evaluation showed superior epithelialization, robust collagen synthesis, and extensive angiogenesis, suggesting a highly effective wound repair process (Figure 9E). The presence of well-organized collagen fibers and increased vascularization further confirmed the enhanced regenerative potential of CP3, making it the most effective treatment in this study.

### 2.13. Immunohistopathological Findings

#### 2.13.1. Immunohistochemical and Histological Evaluation of Wound Healing

**Figure 10:**
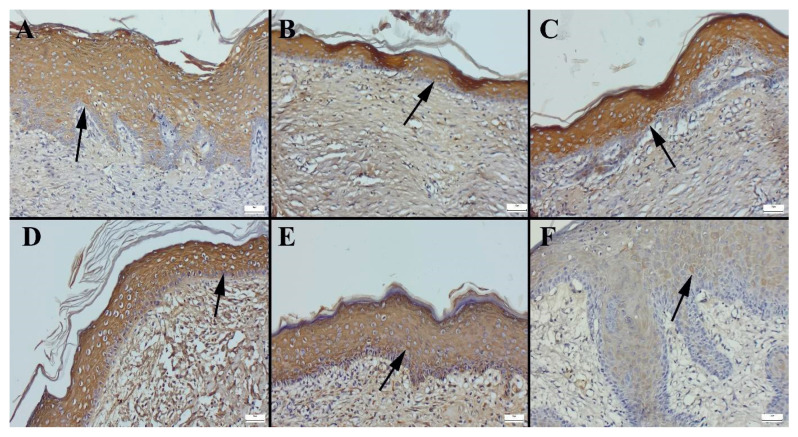
Immunohistochemical expression of cytokeratin according to groups. (**A**) HHPM, (**B**) HHPM-LG8, (**C**) CP1, (**D**) CP2, (**E**) CP3, (**F**) Control; Bars = 50 µm.

The immunohistochemical analysis of cytokeratin expression revealed significant differences between the treatment groups. Cytokeratin, a key marker of epithelial cell differentiation and regeneration, was most prominently expressed in the madecassoside ointment-treated group (CP2). In this group, intense cytokeratin staining was observed in the newly forming epithelial layers at the wound edges, suggesting accelerated epithelialization and enhanced re-epithelialization processes (Figure 10). Conversely, the control group exhibited the lowest cytokeratin expression, indicating delayed epithelial regeneration and a slower wound-healing process. Among the other formulations, notable increases in cytokeratin expression were observed in the groups treated with CP3, CP1, and the hydrogel carrier material, demonstrating their positive effects on epithelialization. The most pronounced cytokeratin expression during wound healing, however, was observed in the liposome-based formulation group. This finding suggests that liposomal formulation played a crucial role in enhancing epithelial cell proliferation and differentiation, potentially due to improved bioavailability and deeper penetration of active compounds.

#### 2.13.2. VEGF Expression and Angiogenesis

**Figure 11:**
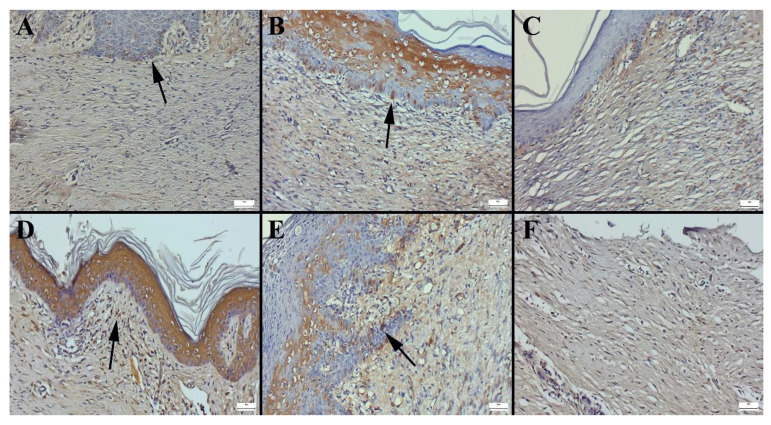
Immunohistochemical expression of VEGF according to groups. (**A**) HHPM, (**B**) HHPM-LG8, (**C**) CP1, (**D**) CP2, (**E**) CP3, (**F**) Control; Bars = 50 µm.

The expression of vascular endothelial growth factor (VEGF), a key regulator of angiogenesis and tissue regeneration, also varied across the treatment groups. Immunohistochemical staining demonstrated differences in VEGF expression in both epithelial and connective tissue cells, with the control group displaying the lowest expression levels. This finding is consistent with the histopathological observations, where the untreated wounds exhibited minimal angiogenesis and delayed tissue repair. In contrast, the CP2-treated group (madecassoside ointment) showed the highest VEGF expression, suggesting a strong pro-angiogenic effect that contributed to enhanced vascularization and tissue regeneration (Figure 11). Similarly, liposomal formulations displayed the highest VEGF expression among the tested preparations, indicating their potential to stimulate neovascularization. The statistical analysis of VEGF expression levels across different groups is summarized in Table 5, further confirming the significant differences between treatment regimens.

#### 2.13.3. Collagen Maturation and Connective Tissue Remodeling

**Figure 12:**
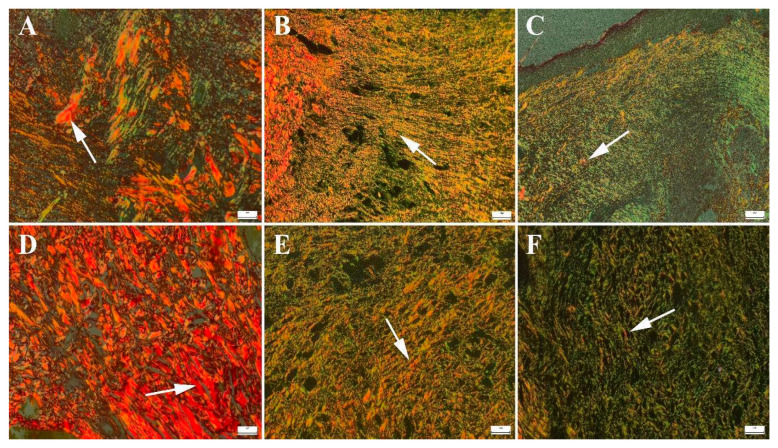
Appearance of Type III and Type I collagen formation according to groups. (**A**) HHPM, (**B**) HHPM-LG8, (**C**) CP1, (**D**) CP2, (**E**) CP3, (**F**) Control; Bars = 50 µm.

Picrosirius red staining revealed distinct patterns of collagen deposition in the defect areas. Fluorescence microscopy analysis showed an increased presence of green fluorescence, indicating Type III collagen, which is characteristic of early stage wound healing and granulation tissue formation (Figure 12). Statistical analysis results were given in Table 7.

## 3. Conclusions

A study of the particle properties of HHPM-loaded liposome formulations prepared with L-S100 revealed that HHPM-L1 and HHPM-L2 formulations exhibited higher particle size and lower zeta potential. These particle properties are hypothesized to be associated with the higher phase transition temperature and non-hydrogenation. PDI values were >0.3, suggesting the possibility of stability issues and coagulation [34]. In contrast, HHPM-L3/4 formulations developed with 90H exhibited reduced dimensions and augmented negative zeta potential values, suggesting that these formulations may possess enhanced physical stability. In addition to these observations, the PDI values of the liposome formulations HHPM-L3 and HHPM-L4 prepared with 90H are <0.3, indicating greater stability.

In relation to the encapsulation efficiency of hypericin in liposome formulations, it was observed that 90H phospholipids with a lower thermal transition temperature encapsulated a higher amount of HHPM when utilized in the same ratio.

In order to understand the encapsulation efficiency of liposome formulations, the peak obtained at a single point in the chromatogram performed at 270 nm in the HPLC chromatogram shows that it is validated in terms of linearity as specified in the ICH Q2 R1 guideline [43]. The hypericin concentration was found to be 0.487% in HHPM-L4, as determined by the equation y = 0.0071x + 0.00126 with R2 = 0.9974. All analytical procedures were conducted in accordance with established protocols.

A comparison of the study’s findings on the spherical structure of liposomes with those cited in the literature reveals a high degree of similarity [44].

The Hixson–Crowell kinetic model hypothesized that the release of the active substance is driven by two mechanisms: diffusion and partitioning from the surface [45]. The model elucidates that the release of hypericin is facilitated by diffusion from the liposomes, while the release from the in-situ gel occurs over a time-dependent trajectory.

In the development of in-situ gel formulations, two distinct concentrations of Poloxamer 188 and Poloxamer 407 were utilized. The GF1 formulation comprises P188/407 at concentrations of 20/10%, which initiates gelation at 22 °C. This observation indicates that the formulation may potentially induce gel formation under ambient conditions [46]. In contrast, the GF2 formulation employs a lower concentration of P188/407, measuring 18/4%. The gel formation temperature of the GF2 formulation was observed to commence at 28 °C. This temperature was hypothesized to be more conducive to the development of formulations designed for wound healing. Furthermore, it was observed that the U30 polymer used in the GF2 formulation exhibited enhanced stability across a range of pH values.

The results of the release study demonstrated that the release of hypericin from liposome-loaded hydrogels occurred over a period of 80 h, with the total amount of released active substance being 83%. The findings of the release studies indicate that the formulated product consistently provides effective treatment [34].

The stability of the HHPM-LG8 formulation was studied over a period of 180 days, during which minimal changes in particle size and zeta potential were observed at a temperature of 4 ± 2 °C (60 ± 5% RH). This indicates that the formulation maintains its structural integrity under these storage conditions. However, at room temperature (25 ± 2 °C; 60 ± 5% RH) and elevated temperatures (25 ± 2 °C; 60 ± 5% RH), there was an increase in particle size, indicating liposome instability at elevated temperatures and emphasizing the necessity for adequate storage conditions.

The zeta potential remained constant between −24.4 ± 3.7 mV and −23.5 ± 4.2 mV, thus confirming colloidal stability. Furthermore, a slight, non-significant decrease in encapsulation efficiency below 10% was observed under refrigerated conditions. However, a significant decrease in encapsulation efficiency was noted at room temperature and higher temperatures, suggesting that liposome performance is compromised in suboptimal environments [43].

The hydrogels demonstrated the capacity to preserve both viscosity and pH values, thereby ensuring stability and compatibility with wound healing processes. In general, the HHPM-LG8 formulation is stable when refrigerated, but elevated temperatures compromise its stability and necessitate appropriate storage conditions for extended use.

The antioxidant activity of the developed HHPM-LG8 formulation was compared with HPPM, CP1, CP2, and CP3 by DPPH, ABTS+, and FRAP assays. In all antioxidant activity studies of liposomal formulations, a significant increase in antioxidant activity was observed compared to HPPM. It has been reported that the antioxidant activity of St John’s wort plant is due to the phenolic compounds in its structure [47]. It has been reported that this activity varies in correlation with rutin, hyperoside, quercetrin, and quercetin compounds in the structure of the plant [48].

The antioxidant potential of ethanol extracts of *Hypericum perforatum* L. was determined by FRAP (3.7 µmol Fe^2+^/mg), DPPH (20.5 EC50 µg/mL), and ABTS (1.02 mmol Trolox/g) methods [49]. It was observed that liposome and HHPM LG8 gel formulations exhibited antioxidant activity similar to that of other commercial products and experimental groups.

As demonstrated in earlier research, these oils have been found to possess antibacterial and anti-biofilm properties, effective against both Gram-positive and Gram-negative bacteria [50]. Hydrogels represent a significant component in the field of wound healing, owing to their capacity for facile shaping, antibacterial properties, and non-toxicity. The present findings thus confirm that the antibacterial activity of the HP-containing liposome formulation is enhanced against *E. coli* and *S. aureus* while maintaining hydrogel stability.

The antimicrobial activity of the HPPM-LG8 formulation was found to be superior to that of conventional and commercial products. The MIC and MBC analyses revealed that HPPM exhibited similar antimicrobial potency against both bacteria (see Table 5). Of particular note is the observation that the liposomal formulation exhibited a significantly higher level of activity in comparison to commercial products such as CP1 and CP2, underscoring its potential as a more efficacious treatment option. These results underscore the efficacy of liposome delivery in enhancing the bioavailability and therapeutic effects of HP, thereby paving the way for the development of advanced antimicrobial herbal formulations for clinical applications.

The results demonstrated that there were significant differences in wound healing scores between the groups. On days 4, 8, and 12, the scores of all groups were found to be significantly different from the control group (*p* < 0.05). This finding suggests that the treatments had a substantial impact on wound healing when compared to the absence of any control treatment. A further observation of the note was the marked differences that were apparent when conventional formulations were compared with hydrogel-based liposome formulations (*p* < 0.05). This finding suggests that hydrogel formulation enhances wound healing efficacy by modulating the performance of the nanoparticles. However, no significant difference was found between the liposome formulations and when compared to CP3, which was used as a positive control. This finding indicates that the HHPM-LG8 formulation, while effective, exhibits a comparable efficacy to CP3, a well-known antibiotic. A notable observation was the presence of significant differences when comparing the commercial HP preparation with HPPM-LG8 (*p* < 0.05, Figure 7 and Figure 8), suggesting that the HHPM-LG8 formulation exhibited superior efficacy in promoting wound healing in comparison to the commercial HP product. These findings underscore the potential of HHPM-LG8 hydrogel formulations for wound healing. However, further optimization is required to achieve efficacy equivalent to or higher than that of conventional antibiotics such as CP3. The results indicate that the HHPM-LG8 formulation is a toxicologically safer, antibiotic-free, and more potent liposome-gel carrier formulation against pathogens when compared to the other groups.

Histopathological analysis of the skin defect areas revealed that epithelialization was slowest in the untreated control group, accompanied by minimal collagen synthesis and limited angiogenesis, indicating poor healing (Figure 9F). Conversely, the CP3 group demonstrated optimal healing outcomes, characterized by superior epithelialization, collagen synthesis, and angiogenesis (Figure 9E). The liposome formulation (Figure 9B) demonstrated significant improvement, comparable to commercial products such as CP1 and CP2 (Figure 9C,D). In comparison to the HHPM or liposome formulations (Figure 9A,B), the liposome formulation demonstrated a substantially more pronounced improvement.

Collagen protein, the most abundant protein in the body, plays a crucial role in the wound-healing process. It is composed of amino acids such as proline and hydroxyproline [51]. Biomarkers are defined as measurable substances that serve as indicators of physiological or pathological processes [52]. Collagen deposition was found to be more prominent in the liposome group (Figure 12B) than in the HHPM group (Figure 12A), as evidenced by Picro Sirius Red staining, which highlighted an increase in type I collagen in these groups. Among the study groups, the CP2 group exhibited the highest collagen synthesis (Figure 12D), followed by the CP3 group (Figure 12E) and the CP2 group (Figure 12C), while the control group (Figure 12F) showed less pronounced collagen synthesis.

The application of HP externally has a long history, spanning centuries, for the treatment of minor burns, wounds, and inflammations. The mechanism of action of HP involves the promotion of wound healing through the stimulation of collagen synthesis, fibroblast formation, and re-epithelialization in a hydrated environment while concurrently reducing transforming growth factor-β (TGF-β) levels to prevent bacterial infection [53]. Studies conducted on the process of wound healing have demonstrated the beneficial effects of HHPM in various stages, including hemostasis, inflammation, proliferation, and remodeling. Clinical success was reported with bio-nanoparticle formulations combining HP and Aloe vera [54]. The present findings lend support to these results by revealing that HHPM-loaded hydrogel liposomes outperformed commercial products by significantly improving healing markers such as wound closure rate and diameter. Furthermore, the high encapsulation efficiency and hypericin concentration of HHPM-LG8 may be pivotal in accelerating wound healing by promoting hemostasis, reducing inflammation, and increasing angiogenesis.

Cytokeratin expression was most prominent in the CP2 group (see Figure 10D), followed by the HHPM-LG8 group (see Figure 10B), the CP3 group (see Figure 10E), and the CP1 group (see Figure 10C). The lowest expression was observed in the control group (Figure 10A). Vascular endothelial growth factor (VEGF) expression was most prominent in the CP2 group (Figure 11D), followed by the HHPM-LG8 (Figure 11B), CP3 (Figure 11E), and CP1 (Figure 11C) groups. The lowest expression was observed in the control group (Figure 11F). VEGF, a critical factor in angiogenesis and wound healing, exhibited a significant increase in the treated groups, suggesting that healing was accelerated [55]. VEGF expression is not detected in healthy skin; however, it is strongly induced in response to cutaneous injury, thus promoting the proliferation of new blood vessels at the site of injury [56]. The investigation revealed that wound healing scores were significantly improved in the liposome group in comparison with the control group. However, no significant difference was observed when the commercial products were compared with each other. In this research, HHPMs were prepared with Lipoid S100 in accordance with recent research trends. The liposome formulations demonstrated favorable outcomes by augmenting the expression of keratin and collagen (see Figure 10). Overall, the HPPM-LG8 formulation containing HHPM exhibited significant potential for wound healing and outperformed conventional formulations both in vitro and in vivo evaluations.

A significant discrepancy was identified when conventional formulations were compared with hydrogel-based liposome formulations (*p* < 0.05). This finding indicates that enhanced formulation modifies the performance of the nanoparticles and potentially enhances wound healing efficacy. However, no significant difference was found between the liposome gel formulations and tetracycline, which was used as a positive control. This finding indicates that liposome gel formulations, while effective, demonstrate comparable performance to tetracycline, a well-known antibiotic. A notable finding emerged from the comparison of the commercial HPM preparation with the HHPM-LG8 formulations (*p* < 0.05, Figure 7 and Figure 8), which revealed significant discrepancies. This finding indicates that HHPM-LG8 formulations are more effective for wound healing than the commercial HPM product. These findings underscore the potential of HHPM-LG8 formulations for wound healing.

## 4. Materials and Methods

### 4.1. Materials

Hypericin, Cholesterol (CHOL), Mueller–Hinton broth, and Mueller–Hinton agar were purchased from Sigma Chemical Co. (St. Louis, MO, USA). All phospholipids, phosphatidylcholine from non-GMO soybean (S 100), and hydrogenated phospholipids from non-GMO soybean (90H) were purchased from Lipoid AG (Ludwigshafen, Germany). Poloxamer 188 and Poloxamer 407 were purchased by BASF (Ludwigshafen, Germany). Carbopol^®^ Ultrez 21 and Carbopol^®^ Ultrez 30 were purchased by Lubrizol Corporation (Wickliffe, OH, USA). *Olive* Oil, *H. perforatum* L. Macerate was purchased by New Raw Material (Antalya, Türkiye). Sabouraud Dextrose Agar and Sabouraud Dextrose broth were purchased from Neogen (Lansing, MI, USA). *Candida albicans* (ATCC 10231) and *Escherichia coli* (ATCC 8739) were used as microorganisms.

All other materials were analytical grade and used without purification.

### 4.2. Preparation of Liposome Dispersions and Liposomal Gel Formulations

The liposome dispersions were prepared via the thin film lipid layer method, as outlined in the relevant literature [10,57]. Briefly, the PC: DCP: CHOL was prepared in the molar ratios indicated in Table 8. In a 100 mL round-bottomed flask, the liposome ingredients with HHPM were dissolved in 20 mL of chloroform–methanol (2:1) organic solvent. Four different liposome formulations were designed; the formulations and codes are given in Table 8.

The organic solvent mixture (chloroform–methanol) was then subjected to rotary evaporation (Heidolph, Germany) at a rotation speed of 150–200 rpm at 40–45 °C under vacuum. The thin film layer formation process was observed by the evaporation of the organic solvent. The hydration process was carried out in 1 mL of ultrapure water for each 1 mg of lipid, 54 ± 2 °C for 90H, and 23 ± 2 °C for S100, considering the phase transformation temperature of lipids. The HHPM was encapsulated using the passive encapsulation method. Utilizing inert glass spheres with a diameter not exceeding 2 mm resulted in the formation of multilayer liposomes. This process occurred through the separation of the thin film from the glass surface.

The extrusion method was used to obtain monodisperse liposomes. In this process, the liposome dispersion was monodispersed by passing through polycarbonate filters, allowing liposomal vesicles to form in the desired size range and homogeneity. The extrusion process was repeated 4 times using a 400 nm and 200 nm polycarbonate membrane filter, respectively.

The liposomal in-situ gel formulations were prepared by incorporating HHPM liposomes into gel carriers. Due to the favorable bioadhesive properties of Carbopol^®^ derivatives, Ultrez 21 (U-21) and Ultrez 30 (U-30) were utilized as gelling agents at a concentration of 0.5% in distilled water [14,38]. Poloxamer 188 and Poloxamer 407 were added to the formulation to ensure in-situ gel formation. Eight different liposomal in-situ gel formulations were prepared by adding four different liposome formulations into two different gel formulations. The composition of the prepared formulation is given in Table 9 and Table 10.

Eight different gel formulations were designed. All formulations are shown in Table 11.

### 4.3. Particle Size, Polydispersity Index, and Zeta Potential

The dynamic light scattering (DLS) analysis was performed to determine the z-mean and polydispersity index (PDI) of the liposome dispersions using a Malvern Zetasizer (Malvern, Worcestershire, UK) (Nano-ZS) [58]. The refractive index for the dispersing medium was set to 1.33 (for water at 25 °C), while that of liposome dispersions was set to 1.45. Samples were diluted in ultra-pure water until the count rate (in kilo counts per second, kcps) fell below 500 and then analyzed in disposable plastic cuvettes. Each sample was analyzed at 25 ± 2 °C with three replicate measurements (n = 3).

### 4.4. Evaluation of the Morphology

The diameter, homogeneity, and morphology of liposomes were examined by scanning electron microscopy (SEM). Initially, the samples were affixed by cryofixation with liquid nitrogen to maintain structural integrity. The protocol for CO_2_-based critical point drying (Leica EM CPD300, Leica, Wetzlar, Germany) was applied, and a 5 nm Au/Pd coating was performed using a Quorum Q150T ES sputter coater (Crawley, East Sussex, UK). Liposomes to a brass stud using double-sided adhesive tape. They were then coated with a thin layer of gold to render them electrically conductive [59]. SEM images were recorded at an accelerating voltage of 10 keV.

### 4.5. Encapsulation Efficiency

The loading amount of hypericin in the HHPM and the encapsulation efficiency were determined using a solvent extraction method under reduced pressure and temperature.

To ascertain the proportion of active ingredient incorporated into the liposome dispersions, ultracentrifugation was performed at 15,000 rpm for 30 min at 4 °C, which resulted in the separation of the mixture into a ‘supernatant’ and a ‘coagulated’ phase [60]. Following the addition of 5% Triton X-100 to the precipitate and its dilution with the mobile phase, the precipitate was passed through a regenerated cellulose filter. The quantification of hypericin in the samples from the above process was then performed using HPLC (Agilent 1100, Santa Clara, CA, USA) based on the previously determined regression equation (R^2^ = 0.9994, y = 157.717 + 189.4329) and the following formula. The hypericin in the HHPM was monitored in the HPLC analysis. The encapsulation efficiency was calculated using Equation (1).

(1) $$E.E.\%=\frac{The\;amount\;of\;Hypericin\;obtained\;as\;a\;result\;of\;the\;analysis\times 100}{Amount\;of\;Hypericin\;added}$$

### 4.6. Rheological Behavior and pH Measurement

The in situ gels GF1-GF2 viscosity measurements were performed by first evaluating temperature and pH variability to examine the effects of individual polymers. The gels were then tested under storage conditions of pH 4/4 °C and pH 7/30 °C conditions, which are suitable for the wound site (Brookfield DV3T HA, Toronto, ON, Canada). Viscosity and shear stresses were recorded at increasing shear rates. The rheological behavior of the formulations was interpreted by obtaining viscosity and shear stress curves for different shear rate values [61]. The viscosity measurements of in-situ liposomal gels were carried out by first evaluating the temperature and pH variability to examine the effects of individual polymers. The viscosity measurements were then tested under storage conditions of pH 4/4 °C and pH 7/30 °C conditions suitable for the wound area.

### 4.7. In Vitro Release Study

The dialysis bag method was utilized in the release study of HHPMs from liposomal gels, with phosphate buffer salt (PBS) pH 7.4 employed as the release medium. At a temperature of 37 ± 0.5 °C, liposomal gels containing active herbal extracts were positioned within a dialysis membrane characterized by a pore width of 2.4 nm and a permeable range of 3–5 kDa molecular weight by sealing both ends. The membrane was hydrated in distilled water for 30 min before use. The dialysis bag was immersed in 30 mL of pH 7.4 PBS [62]. Five hundred microliters of samples were taken at 0.5, 1, 2, 2, 3, 4, 4, 5, 6, 7, 8, 9, 10, 11, 12, 24, 36, 48, 60, 72, and 80 h. The quantification of the samples was performed by HPLC following the necessary dilutions. The release of hypericin from the hypericin-loaded liposomal hydrogel formulation was carried out by HPLC chromatography (Agilent 1100, USA). In the study, analytical validation of hypericin was performed in accordance with ICH Q2 R1 guideline, and a correlation equation y = 157.7117x + 189.4329, R^2^ = 0.9994 was obtained. The RSD values were found to be less than 2, thus validating the study in terms of linearity as per ICH Q2 R1 guidelines. After the collection of each sample, 500 µL of fresh pH 7.4 PBS medium was added. Each sample was repeated on three occasions.

With the results obtained, the drug release kinetics were analyzed by mathematical methods per zero-order, first-order, Higuchi, Korsmeyer-Peppas, and Hixson–Crowell kinetics. The equations of these kinetics are given in Table 12 [63].

Q is the fraction of drug released at time interval t; k, k_0_, k_1_, k_h_, and k_hc_ are the values of the kinetic constants related to Krosmeyer-Peppas, zero-order, first-order, Higuchi, and Hixson–Crowell models, respectively, and n is the diffusion exponent, accounting for the mechanism of drug release.

### 4.8. Antioxidant Activity Study

The ability to inhibit DPPH and ABTS radicals was measured (IC50), with 50% of the extract in the reaction mixture resulting in inhibition. The percentage inhibition rate was calculated by obtaining the amount of hypericin present in the added sample [64]. The antioxidant activity of liposomal gels was determined by DPPH, ABTS+, and FRAP tests [65].

The DPPH (2,2-diphenyl-1-picrylhydrazyl) test was conducted in accordance with the following protocol: solutions of HHPMs and liposomes were prepared in MeOH at 1 mg/mL for the test samples. For the radical activity, a 1 µM/mL DPPH solution was prepared in MeOH. For the test, 100 µL of the DPPH/MeOH solution was added to 200 µL of the sample solutions and incubated in the dark for 30 min. A mixture of 1 mL DPPH solution and ultrapure water was prepared for the control. Absorbance measurement was then carried out with a spectrophotometer at 517 nm against a blank cell. The percentage of scavenging activity of each example on DPPH radical was calculated as %I using the following equation:

DPPH radical scavenging effect (%) = (Control absorbance − Sample absorbance)/(Control absorbance × 100)

The ABTS+ test was conducted by preparing an ABTS+ stock solution, which involved the dissolution of 7 mM (383 mg) ABTS and 66.2 mg (2.45 mM) potassium sulphate in 100 mL methanol. Subsequently, 2 mL of ABTS+ solution was added to 1 mL of the test samples. The samples were then incubated at room temperature in the dark for 10 min. For the control, 2 mL of ABTS+ solution was mixed with ultrapure water, and the measurement was performed. Absorbance measurement was carried out with a spectrophotometer at 734 nm against a blank cell. Samples were prepared on three occasions, and repeated measurements were performed. The percentage of scavenging activity of each example on ABTS^+^ radical was calculated as %I using the equation.

I% = [(C_A_ − S_A_)/C_A_] × 100

where C_A_ is the absorption of control and S_A_ is the absorption of the tested example solution.

In the FRAP test, 0.7 mL samples were extracted from the test samples, and 2.3 mL of FRAP reagent was incubated at 37 °C for 30 min in a dark environment. Absorbance was measured with a spectrophotometer at 593 nm against a blank cell. The standard curve was linear between 25 and 800 mM. Results were expressed as mM TE/g.

### 4.9. Antimicrobial Activity: Microdilution Method

The microdilution method was used for the antimicrobial activity, as recommended by the Clinical and Laboratory Standards Institute [43]. The method used strains of *E. coli* (ATCC 11775) and *Candida albicans* (ATCC 10231) to test for antimicrobial activity. The study was carried out on *H. perforatum* oil, *H. perforatum* oil-loaded liposomal gel formulations, physiological saline (S.F.) was used as the negative control, and CP1, CP2, and CP3 were used as the positive control.

The inoculation of microorganisms was conducted in Mueller–Hinton Agar and Sabouraud Dextrose Agar media, and the appropriate conditions were met. Subsequently, the microbial suspensions were diluted and prepared to a turbidity of 0.5 MacFarland. The test was performed by adding 100 µL of the appropriate medium to each well of a 96-well plate. Subsequently, the formulations were subjected to a seven-fold dilution series, commencing from the final concentration of 500 µg/mL. A total of 100 µL samples were prepared from the first well to the last well at concentrations of 500, 250, 125, 62.5, 31.25, 15.62, 7.81, and 3.90 µg/mL, respectively. Subsequently, 5 µL of microorganism suspensions, prepared in advance, were added to each well. Incubation was performed at 36 ± 1 °C for 18–24 h for bacteria and at 27 ± 1 °C for 48 and 72 h for yeast. At the end of incubation, microbial growth was determined by measuring the absorbance in each well on a plate reader. Absorbance was read at a wavelength of 600 nm for bacteria and 530 nm for yeast using a microplate reader (Biotek Instrument Inc., Highland Park, VT, USA). The last well without growth was considered as the MIC value.

### 4.10. In Vivo Wound Model and Experimental Plan

This study is guaranteed by the Burdur Mehmet Akif Ersoy University Experimental Animal Ethics Committee in accordance with the Reporting of In Vivo Experiments (ARRIVE) 2.0 guidelines with decision number 2020/06. Six different experimental groups were studied, with six 32–36-week-old New Zealand breed rabbits weighing 1000–1500 g in each group. The experimental groups were Groups (A) HHPM, (B) HHPM-LG8, (C) CP1, (D) CP2, (E) CP3, and (F) Control. For each group, the treatments were administered four times a day for a period of 12 days. In the postoperative period, the wounds of the rabbits were measured on days 4, 8, and 12 using calipers. The wound healing rate was calculated using the following formula:

Wound healing rate (%) = (AWi − AWn)/AWi × 100)

In which AWi denotes the initial wound area and AWn denotes the wound area at day n [66].

### 4.11. Histopathological Evaluation

Histopathological examination using two serial sections of 5-micron thickness were taken and stained with hematoxylin and eosin (HE) for general tissue examination under the light microscope. To evaluate connective tissue healing, they were stained with a commercially available kit (ab150681, Abcam, UK) using the Picro Sirius Red method [67].

They were immunohistochemically analyzed using the streptavidin–biotin complex peroxidase method. Immunohistochemical expressions were scored semiquantitatively from 0 to 3 (0: negative, 1: mild, 2: moderate, 3: strongly positive). Microphotography and morphometric examination were performed using the Database Manual Cell Sens Life Science Imaging Software System version 4.2 (Olympus Corporation, Tokyo, Japan), and ImageJ version 1.48 (NIH, Bethesda, MD, USA) was used for score evaluation [68].

### 4.12. Statistical Analysis

Statistical analysis was conducted using the Statistical Package for Social Sciences (SPSS) 22.00 (SPSS Inc., Chicago, IL, USA). Initially, the normality of distribution was assessed using the Shapiro–Wilk test. Since the data demonstrated a normal distribution (*p* > 0.05), comparisons between the groups were made with a one-way analysis of variance (ANOVA). The Duncan test was used to identify differences between groups. For nonparametric data, the Mann–Whitney U test and Dunnett’s C test were used to detect differences between groups. Values of *p* < 0.05 are considered statistically significant. GraphPad software Prism version 10.1.2 was used for the graphs.

## Figures and Tables

**Table 1:** Characteristics of HHPM-loaded liposomes in terms of mean particle size, PDI, zeta potential, and encapsulation efficiency.

Lipoid S100					
**Formulation Code**	**Batch Composition (w/w/w/w)**	**Particle Size (nm) ± SD**	**Polydispersity Index (PDI) ± SD**	**Zeta Potential (mV) ± SD**	**Encapsulation Efficiency of HPM (%) ± SD**
HHPM-L1	L-S100/DCP/Chol/HPM (24:4:12:10) HPM-loaded liposomes	335.1 ± 1.49	0.435± 0.075	−16.2 ± 2.1	84.22 ± 5.19
HHPM-L2	L-S100/DCP/Chol/HPM (28:4:8:10) HPM-loaded liposomes	218.4 ± 1.15	0.339 ± 0.034	−17.1 ± 1.3	89.35 ± 4.51
Lipoid 90H					
**Formulation Code**	**Batch Composition (w/w/w)**	**Particle Size (nm) ± SD**	**Polydispersity Index (PDI) ± SD**	**Zeta Potential (mV) ± SD**	**Encapsulation Efficiency of HPM (%) ± SD**
HHPM-L3	L-90H/DCP/Chol/HPM (24:4:12:10) HPM-loaded liposomes	165.6 ± 2.10	0.314 ± 0.012	−21.2 ± 1.4	85.79 ± 3.02
HHPM-L4	L-90H/DCP/Chol/HPM (24:4:8:10) HPM-loaded liposomes	144.2 ± 0.95	0.247 ± 0.013	−24.2 ± 2.2	91.83 ± 5.62

L-S100: Lipoid S100, DCP: Dihexadecyl phosphate, Chol: cholesterol, HPM: Hypericin in *H. perforatum* L. Macereate, L-90H: Phospholipon 90H.

**Table 2:** Kinetic models of HHPM-L4 (Hypericin).

Kinetic Model		Zero Order	First Order	Higuchi	Hixson–Crowell	Korsmeyer–Peppas
HHPM-L4	Slope	1.2077	−0.0089	4.2405	0.0196	1.309
	Intercept	0.209	2.023	−2.364	0.0024	−0.097
	R	0.991	0.987	0.964	0.992	0.978
	r^2^	0.982	0.976	0.930	0.986	0.957

**Table 3:** Stability results of the gel formulations (n = 3).

**pH**	**Environment Condition**	**Control Period (Month)**	**Appearance**	**Color**	**Odor**	**Viscosity (Mpas)**	**Density (g/cm^3^)**	**Microbiological Result**
3	4 ± 2 °C	0	Homogeneous Gel	Specific	Specific	518 ± 8.4	1.03 ± 0.03	No Growth
	4 ± 2 °C	3	Homogeneous Gel	Specific	Specific	525 ± 9.1	1.04 ± 0.03	No Growth
	4 ± 2 °C	6	Homogeneous Gel	Specific	Specific	537 ± 9.3	1.05 ± 0.04	No Growth
7	4 ± 2 °C	0	Homogeneous Gel	Specific	Specific	43.240 ± 22.1	1.04 ± 0.05	No Growth
	4 ± 2 °C	3	Homogeneous Gel	Specific	Specific	43.440 ± 21.5	1.04 ± 0.06	No Growth
	4 ± 2 °C	6	Homogeneous Gel	Specific	Specific	43.520 ± 23.4	1.05 ± 0.04	No Growth
GF1 Formulation								
**pH**	**Environment Condition**	**Control Period (Month)**	**Appearance**	**Color**	**Odor**	**Viscosity (Mpas)**	**Density** **(g/cm^3^)**	**No Growth**
3	4 ± 2 °C	0	Homogeneous Gel	Specific	Specific	620 ± 7.9	1.06 ± 0.04	No Growth
	4 ± 2 °C	3	Homogeneous Gel	Specific	Specific	625 ± 8.3	1.05 ± 0.04	No Growth
	4 ± 2 °C	6	Homogeneous Gel	Specific	Specific	626 ± 9.1	1.05 ± 0.06	No Growth
7	4 ± 2 °C	0	Homogeneous Gel	Specific	Specific	53.097 ± 17.5	1.06 ± 0.04	No Growth
	4 ± 2 °C	3	Homogeneous Gel	Specific	Specific	53.140 ± 18.7	1.06 ± 0.05	No Growth
	4 ± 2 °C	6	Homogeneous Gel	Specific	Specific	53.170 ± 20.5	1.05 ± 0.04	No Growth
2 Formulation								

**Table 4:** Time-dependent particle size, polydispersity index, and zeta potential values of the HHPM-LG8 formulation at 4 ± 2 °C (60 ± 5% RH) (n = 6).

Code	Time (Day)	Particle Size (nm) ± SD			Zeta Potential (mV) ± SD			PDI ± SD			EE% ± SD		
HHPM-LG8	0	167.84	±	4.17	−24.4	±	3.7	0.238	±	0.007	91.35	±	1.95
	15	168.54	±	5.06	−24.2	±	2.7	0.227	±	0.007	90.01	±	1.99
	30	169.44	±	3.28	−23.9	±	3.6	0.250	±	0.020	88.54	±	1.95
	60	173.90	±	1.72	−23.1	±	0.4	0.196	±	0.040	86.30	±	1.03
	90	178.62	±	3.12	−23.1	±	2.1	0.203	±	0.040	85.30	±	1.03
	180	187.18	±	3.70	−21.9	±	2.4	0.244	±	0.040	83.70	±	1.95

**Table 5:** Time-dependent particle size, polydispersity index, and zeta potential values of the formulation at 25 ± 2 °C (60 ± 5% RH) (n = 6).

Code	Time (Day)	Particle Size (nm) ± SD			Zeta Potential (mV) ± SD			PDI ± SD			EE% ± SD		
HHPM-LG8	0	168.07	±	5.41	−25.8	±	1.3	0.191	±	0.04	91.83	±	1.78
	15	170.02	±	5.73	−24.0	±	3.2	0.204	±	0.030	88.92	±	1.64
	30	171.60	±	4.03	−23.9	±	1.4	0.207	±	0.030	86.20	±	1.18
	60	180.18	±	4.10	−23.8	±	2.5	0.228	±	0.030	84.80	±	0.83
	90	189.93	±	2.11	−23.6	±	1.1	0.237	±	0.03	83.83	±	1.01
	180	215.87	±	1.31	−23.5	±	4.2	0.241	±	0.079	77.30	±	1.01

**Table 6:** MIC and MBC (%) values of *H. Perforatum* L.-containing liposome in situ hydrogel formulation against various strains.

Preparations	Antibacterial Activity µg/mL			
	*E. coli*		*C. albicans*	
	MIC	MBC	MIC	MBC
Olive oil	≥500	≥500	≥500	≥500
HHPM	≥125	≥125	≥125	≥125
In-situ gel	≥500	≥500	≥500	≥500
HHPM-LG8	≥31.25	≥62.5	≥62.5	≥62.5
CP1	≥250	≥250	≥250	≥250
CP2	≥62.5	≥125	≥62.5	≥125
CP3	≥2.6	≥2.6	≥2.6	≥2.6
Negative Control (S.F.)	≥1000	≥1000	≥1000	≥1000

**Table 7:** Statistical analysis results of immunohistochemical scores according to the groups.

Group	Cytokeratin	VEGF
HHPM	2.16 ± 1.16 ^bcd^	1.66 ± 0.51 ^d^
HHPM-LG8	2.83 ± 0.40 ^cd^	3.00 ± 0.00 ^e^
CP1	2.00 ± 0.63 ^abc^	1.16 ± 0.75 ^bcd^
CP2	3.00 ± 0.00 ^d^	3.00 ± 0.00 ^e^
CP3	2.16 ± 0.75 ^bcd^	2.33 ± 0.51 ^e^
Control	1.00 ± 0.63 ^a^	0.33 ± 0.21 ^a^

^a^ HHPM, ^b^ HHPM-LG8, ^c^ CP1, ^d^ CP2, ^e^ CP3.

**Table 8:** Ingredients and codes of liposome formulations.

Formulation Code	Ingredients	Molar Ratio
HPM-L1	S100:DCP:CHOL	60:10:30
HPM-L2	S100:DCP:CHOL	70:10:20
HPM-L3	90H:DCP:CHOL	60:10:30
HPM-L4	90H:DCP:CHOL	70:10:20

**Table 9:** Ingredients and codes of in-situ hydrogel formulations.

Ingredients	GF1 (Conc.%)	GF2 (Conc.%)
Ultrez 21	0.5	-
Ultrez 30	-	0.5
Poloxamers 188	20	18
Poloxamers 407	10	4

**Table 10:** Ingredients of liposomal in-situ hydrogel formulations.

Ingredients	%
HHPM-containing liposome	1
Antimicrobial Preservative	0.95
Propylene glycol	5
Glycerin	2
Carbopol derivatives(U-21/U-30)	0.5
Poloxamers 188	18–20
Poloxamers 407	4–10
Triethanolamine (TEA)	0.45
Ultrapure water	qs. 100

**Table 11:** Liposome in-situ gel formulations and codes.

Formulation Code	I Ingredients
HHPM-LG1	U-21 (0.5%) + Poloxamers (30%) + HHPM-L1
HHPM-LG2	U-21 (0.5%) + Poloxamers (30%) + HHPM-L2
HHPM-LG3	U-21 (0.5%) + Poloxamers (30%) + HHPM-L3
HHPM-LG4	U-21 (0.5%) + Poloxamers(30%) + HHPM-L4
HHPM-LG5	U-30 (0.5%) + Poloxamers(22%) + HHPM-L1
HHPM-LG6	U-30 (0.5%) + Poloxamers(22%) + HHPM-L2
HHPM-LG7	U-30 (0.5%) + Poloxamers(22%) + HHPM-L3
HHPM-LG8	U-30 (0.5%) + Poloxamers(22%) + HHPM-L4

**Table 12:** Mathematical equations of kinetic models.

Kinetic Model	Equation
Zero-order	Q = k_0_t
First-order	ln(1 − Q) = −k_1_t
Higuchi	Q = k_h_t^1/2^
Korsmeyer-Peppas	Q = kt^n^
Hixson–Crowell	Q^1/3^ − W_t_ = k_hc_t

## Data Availability

The original contributions presented in this study are included in the article. Further inquiries can be directed to the corresponding author.
